# Supplementary material for: Improvement of Stable Restorer Lines for Blast Resistance through Functional Marker in Rice (Oryza sativa L.)
Source: Genes (Basel). 2020 Oct 27;11(11):1266. doi: 10.3390/genes11111266 (PMC7692511; doi:10.3390/genes11111266)
Supplement: Supplementary file 1 [file genes-11-01266-s001.pdf]

Supplementary Table S1. List of microsatellite markers polymorphic between donor parent B 95 and recurrent parents CB 87 R and CB 174 R lines. (Marker source: https://archive.gramene.org/markers/microsat/ssr.html)

Chromosome	Total no. of markers analyzed	Total no. of polymorphic markers	Name of the polymorphic markers
1	19	7	RM8077, RM7466, RM403,RM 431, RM 443,RM579, RM8110,
2	14	6	RM 305,RM 427,RM525, RM6290, RM498, RM475
3	10	6	RM1164, RM426,RM 3204,RM 6959,RM 1940,RM3117
4	10	5	RM551, RM3306, RM1236,RM 4405.RM 5686
5	13	5	RM1237, RM305, RM153,RM3575,RM3685
6	14	5	RM1163, RM3414, RM461,RM8226,RM 340
7	20	5	RM542, RM214, RM11, RM134,RM 1268
8	14	7	RM6008, RM6863, RM223, RM477, RM256,RM5808,RM447
9	10	4	RM2190, RM2482,RM3919,RN1896
10	13	7	RM7217, RM6150, RM6128, RM8210, RM229,RM 258,RM3152
11	12	7	RM3605, RM5558, RM1761, RM144, RM441,RM3717,RM1124
12	7	6	RM491, RM101, RM5341, RM3103, RM22,RM3331
**Total**	**156**	**70**	

Supplementary Table S2. Details of blast resistance (*Pi54*), fertility restorer genes (*Rf3*, *Rf4*) in backcross generation

**S. No**	**Generation**	**Total no of plants screened**	**No of plants conformed blast genes *(Pi54)***	**No of plants conformed fertility restoration genes *(Rf3, Rf4)***	**Pyramided gene combination in the conformed lines *(Pi54 +Rf3+Rf4)***
1	F_1	106- 87 R,<br>103-174 R	17- 87 R<br>12- 174 R	-	-
2	BC_1F_1	183- 87 R<br>194 -174 R	21- 87 R<br>17- 174 R	-	-
3	BC_2F_1	173- 87 R<br>191-174 R	14- 87 R<br>17- 174 R	-	-
4	BC_3F_1	160- 87 R<br>183-174 R	12- 87 R<br>15- 174 R		
5	BC_3F_2	210- 87 R<br>260-174 R	41- 87 R<br>46-174 R	9- R, 21-NR, 11- H<br>13-R, 25-NR, 8-H	9-R<br>13-R

Supplementary Table S3. Analysis of Variance for agronomic performance of pyramided lines

Source of Variation	SS	df	MS	F	P-value	F crit
Rows	774.85	25	30.99	1.09	0.37	1.59*
Columns	284765.21	2	56953.04	1999.77	2E-117	2.29*
Error	3559.98	125	28.47			
Total	289100.03	155				

*significance at 5 % level of significance.

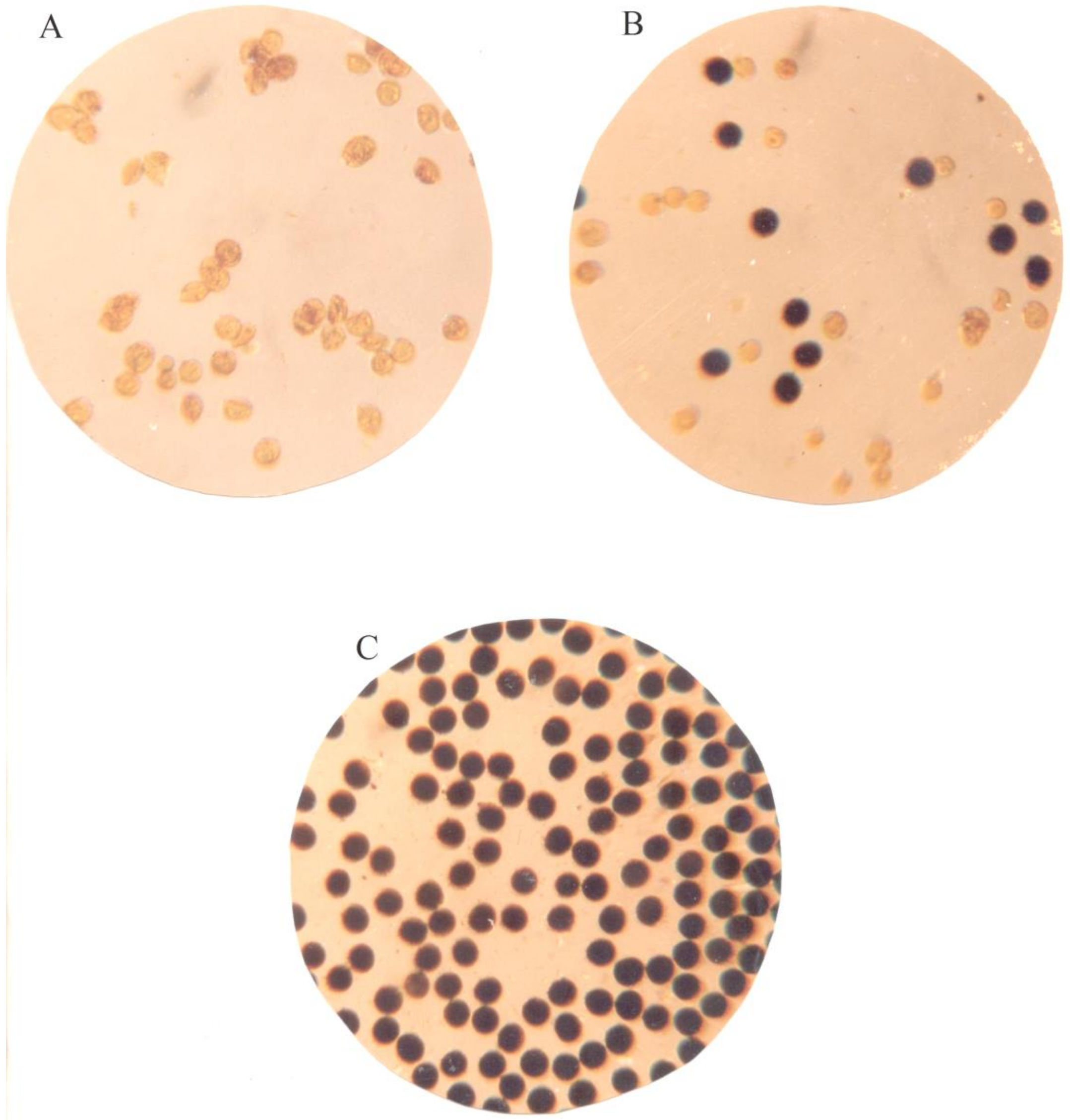

Supplementary Figure S1. Pollen fertility studies on BC_3F_3 plants. **(A)** Sterile. (**B**) Partial fertile /sterile. (**C**) Fertile.

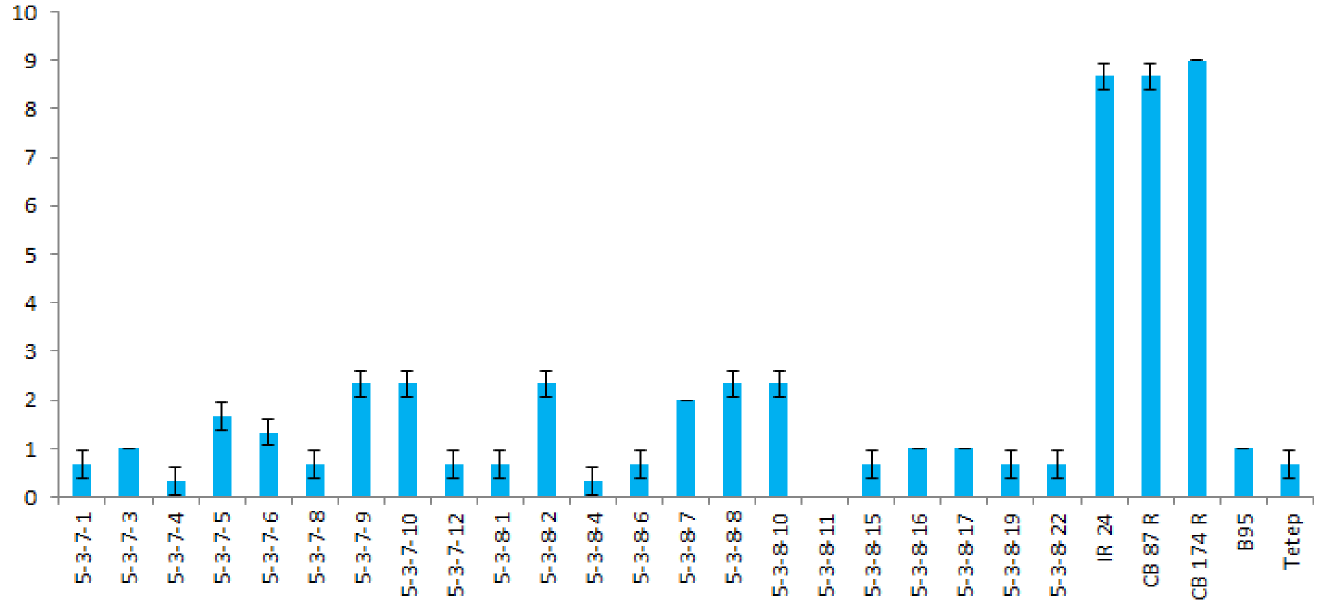

Supplementary Figure S2. Distribution of parents (CB 87 R and CB 174 R) and improved genotypes means for blast response. Tetep and IR 24 are the positive and negative check respectively. B 95 is the donor parent.

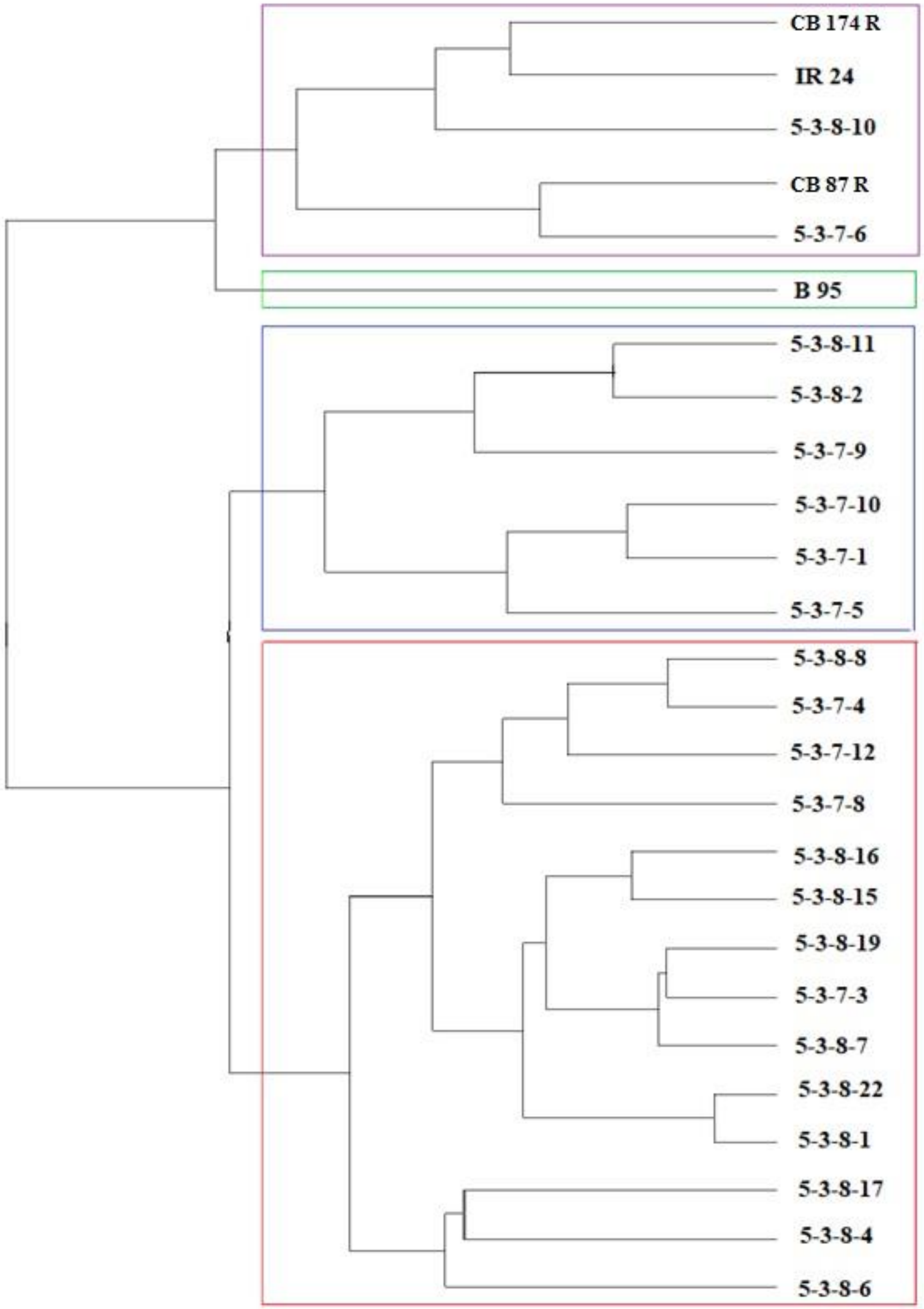

Supplementary Figure S3. Agglomerative clustering method based on seven quantitative characters in pyramided lines
